# Low-normal hemoglobin levels and anemia are associated with increased risk of end-stage renal disease in general populations: A prospective cohort study

**DOI:** 10.1371/journal.pone.0215920

**Published:** 2019-04-25

**Authors:** Sang-Wook Yi, Sung Jin Moon, Jee-Jeon Yi

**Affiliations:** 1 Department of Preventive Medicine and Public Health, Catholic Kwandong University College of Medicine, Gangneung, Republic of Korea; 2 Department of Internal Medicine, Catholic Kwandong University College of Medicine, International St. Mary's Hospital, Incheon, Republic of Korea; 3 Institute for Occupational and Environmental Health, Catholic Kwandong University, Gangneung, Republic of Korea; Postgraduate Medical Institute, INDIA

## Abstract

**Background:**

The impact of low-normal hemoglobin (Hb) levels and anemia on the risk of end-stage renal disease (ESRD) in general populations has rarely been examined.

**Methods:**

510,620 Korean adults aged 40–80 years without known chronic kidney disease (CKD) underwent health examinations during 2002–2003 and were followed-up until 2013. Incidence of ESRD was identified by hospital discharge and clinical visit records.

**Results:**

During a mean follow-up of 10.5, 575 women and 1047 men were diagnosed with ESRD. Lower Hb levels were associated with an increased risk of ESRD at given severity of albuminuria and at given estimated glomerular filtration rate (eGFR). Hb 13–13.9 g/dL in men, Hb 11–11.9 g/dL in women, and trace albuminuria assessed by dipstick urinalysis were associated with more than doubled risk. The risk associated with lower Hb was stronger in older (≥60 years) than younger women. Among 349,993 participants with information on eGFR, the multivariable-adjusted HRs associated with 1 g/dL lower Hb in participants with eGFR values ≥60, 30–59, and <30 mL/min/1.73 m^2^ were 1.34 (95% CI, 1.17–1.54), 1.55 (1.38–1.74), and 1.75 (1.47–2.09), respectively (*P*interaction between eGFR groups = .06).

**Conclusions:**

Low-normal Hb levels and anemia are risk factors for ESRD incidence in person without CKD and for CKD progression to ESRD. Lower Hb increases the risk of ESRD through synergistic biological interactions with lower eGFR and albuminuria. The impacts of lower Hb may be stronger in older than younger women. Proper management and screening at earlier stage of Hb decline and anemia might reduce the burden of CKD.

## Introduction

Approximately 1.2 million people died of chronic kidney disease (CKD) and subsequent end-stage renal disease (ESRD) worldwide in 2015, a 31.7% increase from 2005, and these diseases rose in rank to become the leading cause of premature death from 1990 and 2005 to 2015 [1]. Early detection and primary prevention in high-risk individuals are strategies used to reduce the burden of CKD. However, since most patients are asymptomatic until the advanced stages of CKD, determination of who should be screened and who is at high risk in asymptomatic adults has been a challenge [2].

Few studies examined the impact of low-normal hemoglobin (Hb) levels and anemia on development of ESRD, despite the fact that low Hb levels *per se* may contribute to the progression of CKD to ESRD [3,4], while Hb decline and anemia may occur as a consequence of CKD even at early stages [5]. Low-normal Hb levels, such as 13–13.9 g/dL in men, little attention has been paid to. Furthermore, it has been rarely examined and is unclear whether low-normal Hb levels and anemia increase ESRD incidence in individuals with normal kidney function, namely no albuminuria and normal glomerular filtration rate (GFR). A better understanding of the impact of low-normal Hb and anemia on ESRD may help identify high-risk groups and target Hb levels for the prevention, surveillance, and management of CKD in both general populations and CKD patients.

In this prospective cohort study, ESRD incidence was assessed according to Hb levels and albuminuria measured by the dipstick test, and the interaction between Hb levels and albuminuria was examined. In individuals with information on serum creatinine levels, these associations were examined after further consideration of the estimated GFR (eGFR).

## Methods

### Study population and follow-up

Ninety-seven percent of Koreans are insured through the National Health Insurance Service (NHIS) [6]. The study cohort (n = 514,795) was a random sample of the 5.15 million NHIS beneficiaries aged 40–79 years in 2002 who received a health examination during 2002–2003. From this sample, 3789 persons with missing information on cardiometabolic factors including fasting glucose and body mass index (BMI), an extreme BMI (<12 or ≥50 kg/m^2^), or extreme anemia (<7 g/dL) were excluded, as were 386 subjects with known prevalent CKD at baseline. We followed the remaining 510,620 individuals until December 31, 2013 via record linkage to the NHIS data. All patients who were discharged from the hospital due to ESRD or had at least 2 consecutive outpatient clinical visits for ESRD within a 1-year period for the first time during follow-up were considered incident cases. ESRD cases were identified using International Classification of Diseases, 10th revision (ICD-10) codes: N180 during 2002–2010, and N185 during 2011–2013. Due to the possibility of undiagnosed asymptomatic CKD at baseline, this study focused on incidence of ESRD rather than CKD. The authors were granted access to the anonymized data by the NHIS, according to Korean law [7]. This study was approved by the Institutional Review Board of Catholic Kwandong University (CKU-16-01-0301). Informed consent was waived because the data provided by the NHIS have been anonymized according to the strict confidentiality guidelines.

### Data collection

Albuminuria was assessed by the dipstick test, generally using automated strip analysis, and it was reported as negative, trace, 1+, 2+, 3+, and 4+ [8] in baseline health examinations during 2002–2003. Dipstick urinalysis is highly sensitive to albumin and has reasonably good sensitivity and specificity for detecting albuminuria [9–12]. Serum Hb was measured using the cyanmethemoglobin method. Fasting serum glucose and total cholesterol were assayed using enzymatic methods [13]. Blood pressure was measured using a standard mercury sphygmomanometer, with the subject in a seated position. BMI was calculated as measured weight divided by the square of measured height (kg/m^2^)[6]. Smoking status, alcohol use, and history of cancer, heart disease, or stroke were assessed via a questionnaire. We considered individuals to have known prevalent CKD (N18), hypertension (I10-I15), and diabetes (E10-E14) at baseline if they visited a medical institution for those diagnosed conditions at least once within 6 months before or 2 months after the baseline health examination date.

The health examinations and data collection followed a standard protocol officially documented by government. The external quality assessment of clinical chemistry and urinalysis were regularly performed [14,15].

### Statistical analysis

Poisson regression was performed to calculate sex- and age-adjusted ESRD incidence. The hazard ratios (HRs) for ESRD incidence were calculated using Cox proportional hazards models stratified by age (years) at baseline (40–44, 45–54, 55–64, 65–74, and 75–80). The multivariable analysis adjusted for age at baseline (continuous variable), sex, smoking status, alcohol use, BMI, physical activity (at least once a week; yes or no), income status (deciles; <4, 4–7, 8–10 [high income]), dipstick hematuria, and dipstick albuminuria, as well as comorbid diabetes, hypertension, cancer, and heart disease or stroke.

We considered eGFR in 349,993 participants with information on serum creatinine during 2009–2010 health examinations but without known CKD (N18) or extreme eGFR (<10 mL/min/1.73 m^2^). The CKD Epidemiology Collaboration (CKD-EPI) Creatinine equation was used to calculate eGFR [16].

Hb levels were primarily categorized into 5 sex-specific groups (<10, 10–10.9, 11–11.9, 12–12.9, and ≥13 g/dL in women; <12, 12–12.9, 13–13.9, 14–14.9, and ≥15 g/dL in men) in the main analyses, and into 3 groups (<11, 11–11.9, ≥13 g/dL in women; <13, 13–13.9, and ≥14 g/dL in men) in the analyses with consideration of eGFR. Hb levels were also used as a continuous variable. In the main analysis, men and women were analyzed separately, and a subgroup analysis by age (<60 and ≥60 years) was also performed.

The statistical interaction tests that examined the difference in effect size of Hb between subgroups according to sex, age, albuminuria, and eGFR categories used an inverse-variance weighted average method. Subgroup analyses were also used as a sensitivity test. All *P* values were 2-sided. All analyses used SAS version 9.4 (SAS Institute Inc., Cary, NC, USA).

## Results

### General characteristics

During the mean follow-up of 10.5 years, among 233,497 women and 277,123 men, 575 women and 1072 men were diagnosed with ESRD. The mean±SD Hb level was 12.9±1.2 g/dL in women, and 14.8±1.1 g/dL in men. Individuals with lower Hb were more likely to have a low BMI and total cholesterol, as well as severe albuminuria (Table 1). People with severe albuminuria tended to have a high BMI, high total cholesterol, and comorbid diabetes, hypertension, and heart disease/stroke (Table A in S1 File). The individuals with the lowest Hb were both the youngest and the oldest age groups in women, while they were the oldest in men (Fig A in S1 File).

**Table 1:** Characteristics of participants according to sex and Hb categories.

		Women							Men						
	Hb group (g/dL)	Subtotal		≥13	12–12.9	11–11.9	10–10.9	<10	Subtotal		≥15	14–14.9	13–13.9	12–12.9	<12
Variables	Characteristics	N = 233,497		n = 116,459	n = 81,339	n = 24,658	n = 6,884	n = 4,157	N = 277,123		n = 131,107	n = 92,508	n = 40,784	n = 9,700	n = 3,024
Age	years	54.0	±9.9	54.2±9.6	54.2±10.0	54.0±10.6	51.5±10.3	48.3±8.5	52.3	±9.5	50.7±8.7	52.4±9.4	54.9±10.2	58.4±10.8	60.2±11.0
Body mass index	kg/m^2^	24.0	±3.1	24.3±3.1	23.8±3.0	23.4±3.0	23.3±3.0	23.4±3.0	24.0	±2.8	24.5±2.8	23.8±2.8	23.3±2.9	22.6±2.9	22.1±3.1
Total cholesterol	mg/dL	203	±39	207±40	201±38	195±38	191±39	181±35	199	±38	203±38	197±37	192±38	185±38	176±40
Smoking status	Never smoker	216,135	(92.6)	92.2	92.9	93.1	93.3	93.1	111,678	(40.3)	38.0	41.2	43.9	45.7	46.4
	Past smoker	2,152	(0.9)	1.0	0.9	0.8	0.9	0.9	41,262	(14.9)	14.7	15.4	14.4	13.9	14.2
	Current smoker	6,398	(2.7)	3.1	2.5	2.3	2.1	1.8	111,450	(40.2)	42.7	38.8	37.0	35.4	34.4
	Missing	8,812	(3.8)	3.8	3.7	3.9	3.7	4.2	12,733	(4.6)	4.6	4.6	4.7	4.9	5.0
Alcohol use,	None	187,901	(80.5)	80.0	80.9	81.5	80.9	79.9	95,540	(34.5)	31.4	35.1	39.6	44.8	49.2
times/week	<2	35,687	(15.3)	15.7	15.0	14.3	15.2	15.9	124,181	(44.8)	47.5	44.8	39.8	34.6	30.0
	3–7	4,231	(1.8)	2.0	1.7	1.5	1.7	1.5	53,497	(19.3)	19.8	18.8	19.1	18.6	18.7
	Missing	5,678	(2.4)	2.4	2.4	2.7	2.2	2.7	3,905	(1.4)	1.4	1.3	1.5	2.0	2.1
Physical activity	≥1 times/week	74,782	(32.0)	32.2	32.2	31.5	30.7	29.2	135,293	(48.8)	50.2	49.3	46.0	40.7	37.8
Income status,	<4 (low income)	67,202	(28.8)	28.6	28.7	29.8	28.9	29.4	50,306	(18.2)	16.2	18.4	21.6	24.8	26.0
decile	4–7	75,124	(32.2)	32.5	32.1	31.6	31.7	29.1	91,149	(32.9)	31.6	33.1	35.3	37.4	35.0
	>7 (high income)	91,171	(39.0)	38.9	39.2	38.6	39.4	41.5	135,668	(49.0)	52.2	48.4	43.2	37.8	39.1
Age group	<60 years	160,118	(68.6)	68.5	67.6	67.0	75.9	87.8	209,137	(75.5)	81.7	75.1	64.6	50.7	43.4
	≥60 years	73,379	(31.4)	31.5	32.4	33.0	24.1	12.2	67,986	(24.5)	18.3	24.9	35.4	49.3	56.6
Albuminuria	No	225,173	(96.4)	96.3	96.7	96.6	95.7	95.8	267,344	(96.5)	96.2	97.0	96.6	95.2	93.4
	Trace	3,688	(1.6)	1.6	1.5	1.5	2.0	1.3	4,294	(1.5)	1.6	1.4	1.5	1.8	1.8
	1+	3,172	(1.4)	1.5	1.2	1.2	1.5	1.6	3,603	(1.3)	1.5	1.0	1.2	1.9	2.1
	2+	1,157	(0.5)	0.5	0.5	0.4	0.6	1.0	1,460	(0.5)	0.6	0.4	0.5	0.8	2.0
	≥3+	307	(0.1)	0.1	0.1	0.1	0.2	0.4	422	(0.2)	0.1	0.1	0.2	0.3	0.7
Hematuria	No	204,064	(87.4)	88.0	87.1	85.6	87.4	87.8	264,861	(95.6)	95.9	95.6	95.0	93.7	92.4
	Trace	7,482	(3.2)	3.1	3.3	3.7	3.5	3.0	3,554	(1.3)	1.2	1.3	1.3	1.6	1.7
	1+	12,225	(5.2)	5.1	5.3	5.8	4.6	4.5	5,324	(1.9)	1.8	1.9	2.2	2.6	3.2
	2+	6,221	(2.7)	2.5	2.8	3.1	2.5	2.6	2,234	(0.8)	0.7	0.8	0.9	1.4	1.6
	≥3+	3,505	(1.5)	1.3	1.6	1.9	2.0	2.1	1,150	(0.4)	0.3	0.4	0.5	0.8	1.1
Comorbidity	Diabetes^a^	22,906	(9.8)	11.0	8.9	8.4	7.9	6.4	34,362	(12.4)	12.4	11.6	12.9	15.5	19.7
	Hypertension^b^	75,715	(32.4)	35.9	30.0	28.1	24.9	20.1	96,350	(34.8)	35.6	33.5	34.2	37.4	39.2
	Heart disease or stroke^c^	4,858	(2.1)	2.2	2.0	2.0	1.8	1.4	4,736	(1.7)	1.5	1.6	2.1	2.8	3.2
	Cancer^c^	1,604	(0.7)	0.6	0.7	0.8	0.8	0.8	1,258	(0.5)	0.2	0.4	0.7	1.8	3.8

Hb, hemoglobin

Data are expressed as mean ± standard deviation, n(%), or %.

*P* values, which were calculated by the chi-square test and 1-way analysis of variance between Hb groups, were < .001 for each variable, except for comorbid cancer in women (*P* = .006)

^a^Persons with known diabetes by hospital visit records or fasting glucose level ≥126 mg/dL at baseline health screening.

^b^Persons with known hypertension by hospital visit records or systolic blood pressure ≥140 mm Hg at baseline health screening.

^c^Persons with a history of the diseases via questionnaire

### Associations of Hb and albuminuria with ESRD incidence

**Fig 1:**
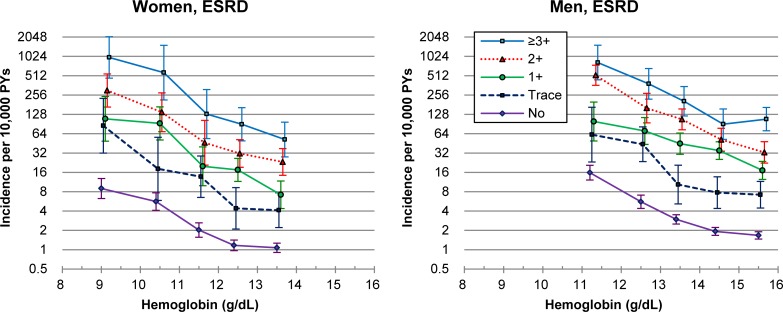
Age-adjusted* incidence of end-stage renal disease (ESRD) in 25 combined hemoglobin and albuminuria groups in women and men. *Poisson regression was used.

A similar reverse-J-curve association of Hb and a linear association of albuminuria with ESRD incidence were found in both men and women (Fig B in S1 File). The age-adjusted incidence of ESRD was generally higher at lower levels of Hb and with more severe albuminuria in each sex (Fig 1). The age-adjusted incidence per 10,000 person-years was 1.07 and 1.67 in men and women with no albuminuria and the highest Hb levels, respectively, while it was 986.1 and 820.6 in men and women with albuminuria ≥3+ and the lowest Hb levels, respectively.

In the multivariable analysis, the pattern of HRs was generally similar to that of age-adjusted incidence. The risk associated with the lowest Hb category and no albuminuria was greater than the risk associated with the highest Hb category and albuminuria of 1+ (Table 2).

**Table 2:** HRs^a^ for ESRD incidence in men and women in 25 combined Hb and albuminuria groups.

	Women						Men					
Albuminuria (dipstick)	Hb group (g/dL)	No. of subjects	No. of cases	*P*-value	HR	(95% CI)	Hb group (g/dL)	No. of subjects	No. of cases	*P*-value	HR	(95% CI)
None	≥13	112,142	145		1.00	(Reference)	≥15	126,138	223		1.00	(Reference)
	12–12.9	78,635	112	.084	1.24	(0.97–1.59)	14–14.9	89,734	197	.042	1.22	(1.01–1.48)
	11–11.9	23,826	59	< .001	2.29	(1.69–3.11)	13–13.9	39,412	147	< .001	1.86	(1.51–2.30)
	10–10.9	6,589	39	< .001	6.29	(4.40–8.98)	12–12.9	9,236	72	< .001	3.47	(2.64–4.55)
	<10	3,981	30	< .001	11.16	(7.46–16.70)	<12	2,824	60	< .001	10.40	(7.74–13.97)
Trace	≥13	1,879	10	< .001	3.21	(1.68–6.11)	≥15	2,135	17	< .001	3.04	(1.85–4.99)
	12–12.9	1,238	7	< .001	4.09	(1.91–8.74)	14–14.9	1,327	12	< .001	3.78	(2.11–6.76)
	11–11.9	382	7	< .001	12.91	(6.03–27.62)	13–13.9	607	8	< .001	5.05	(2.49–10.25)
	10–10.9	136	3	< .001	17.97	(5.70–56.63)	12–12.9	170	10	< .001	17.20	(9.06–32.64)
	<10	53	4	< .001	139.09	(50.98–379.53)	<12	55	4	< .001	23.17	(8.58–62.57)
1+	≥13	1,710	16	< .001	4.96	(2.95–8.34)	≥15	1,914	37	< .001	5.54	(3.90–7.87)
	12–12.9	985	23	< .001	14.17	(9.08–22.11)	14–14.9	962	39	< .001	11.65	(8.24–16.48)
	11–11.9	308	8	< .001	17.32	(8.46–35.45)	13–13.9	478	27	< .001	15.86	(10.57–23.78)
	10–10.9	103	11	< .001	54.91	(29.50–102.21)	12–12.9	186	17	< .001	23.96	(14.53–39.52)
	<10	66	6	< .001	120.44	(52.69–275.30)	<12	63	8	< .001	24.73	(12.09–50.56)
2+	≥13	577	17	< .001	14.58	(8.77–24.23)	≥15	729	26	< .001	9.39	(6.22–14.16)
	12–12.9	384	16	< .001	20.49	(12.16–34.53)	14–14.9	367	23	< .001	16.45	(10.61–25.50)
	11–11.9	110	6	< .001	31.50	(13.79–71.95)	13–13.9	224	28	< .001	31.98	(21.35–47.90)
	10–10.9	44	8	< .001	81.58	(39.78–167.27)	12–12.9	79	14	< .001	40.33	(23.16–70.25)
	<10	42	11	< .001	272.59	(145.61–510.29)	<12	61	31	< .001	136.43	(92.10–202.09)
≥3+	≥13	151	10	< .001	28.84	(15.10–55.10)	≥15	191	22	< .001	29.48	(18.93–45.89)
	12–12.9	97	11	< .001	51.39	(27.46–96.18)	14–14.9	118	13	< .001	22.44	(12.72–39.58)
	11–11.9	32	5	< .001	146.39	(59.64–359.34)	13–13.9	63	14	< .001	46.88	(27.00–81.38)
	10–10.9	12	4	< .001	435.83	(157.67–1204.72)	12–12.9	29	13	< .001	88.17	(49.61–156.70)
	<10	15	7	< .001	810.56	(375.01–1751.97)	<12	21	10	< .001	163.25	(85.07–313.29)

CI, confidence interval; ESRD, end-stage renal disease: Hb, hemoglobin; HR, hazard ratio

^a^ Adjustment for age at baseline, smoking status, alcohol use, physical activity, income status, diabetes, hypertension, a history of heart disease or stroke, a history of cancer, dipstick hematuria, total cholesterol, and body mass index.

In the analysis of 15 combined Hb-albuminuria groups, the associations of lower Hb with ESRD generally did not weaken at ages ≥60 years in comparison to younger ages (Fig C in S1 File). Assuming a linear association, women had greater inverse associations with Hb than men (Table 3; HR per 1 g/dL lower Hb = 1.69 in women versus 1.52 in men, *P*_interaction_ = 0.006). In women, but not in men, the HRs associated with lower Hb were stronger at ages ≥60 years than at ages <60 years. Additionally, the effect size of 1 g/dL lower Hb was not different according to 5 albuminuria categories in both men and women (Table B in S1 File)

**Table 3:** HRs^a^ per 1 g/dL decrease in Hb or per 1 category severe albuminuria for ESRD incidence by sex and age.

	**Women**					**Men**					
**Age group**	**No. of cases**	***P*-value**	**HR per 1 g/dL lower Hb (95% CI)**		***P***_Interaction_ (age)	**No. of cases**	***P*-value**	**HR per 1 g/dL lower Hb (95% CI)**		***P***_Interaction_ (age)	***P***_interaction_ (sex)
All ages	575	< .001	1.69	(1.59–1.79)		1072	< .001	1.52	(1.46–1.59)		.006
40–59 years	266	< .001	1.51	(1.39–1.64)	< .001	548	< .001	1.52	(1.43–1.61)	0.977	.902
60–80 years	309	< .001	1.99	(1.83–2.16)		524	< .001	1.52	(1.43–1.61)		< .001
			**HR per 1 category severe albuminuria (95% CI)**					**HR per 1 category severe albuminuria (95% CI)**			
All ages	575	< .001	2.48	(2.33–2.64)		1072	< .001	2.23	(2.13–2.34)		.008
40–59 years	266	< .001	2.69	(2.46–2.94)	0.017	548	< .001	2.30	(2.15–2.46)	0.226	.005
60–80 years	309	< .001	2.31	(2.11–2.53)		524	< .001	2.17	(2.02–2.32)		.286

CI, confidence interval; ESRD, end stage renal disease; Hb, hemoglobin; HR, hazard ratio; *P*_interaction_ (age), *P*-value for interaction between age groups; *P*_interaction_ (sex), *P*-value for interaction between sexes.

^a^ Adjustment for age at baseline, sex, smoking status, alcohol use, physical activity, income status, diabetes, hypertension, a history of heart disease or stroke, a history of cancer, dipstick hematuria, total cholesterol, and body mass index.

### Analysis after further consideration of the eGFR

**Fig 2:**
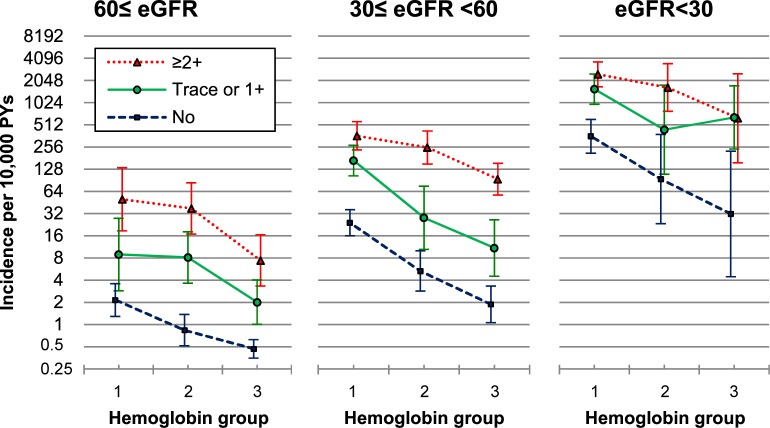
Age-adjusted* incidence of ESRD in 27 combined hemoglobin, albuminuria, and eGFR groups. ESRD, end-stage renal disease; eGFR, estimated glomerular filtration rate using the Chronic Kidney Disease Epidemiology Collaboration (CKD-EPI) creatinine equation. Hemoglobin groups 1, 2, and 3 correspond to <11, 11–11.9 and ≥12 g/dL, respectively, in women, and <13, 13–13.9 and ≥14 g/dL, respectively, in men. *Poisson regression was used.

Among 349,993 participants with information on eGFR, during the mean follow-up of 4.0 years, 316 individuals were diagnosed with ESRD. Individuals with a lower eGFR tended to be older, female, and to have comorbid diabetes and hypertension (Table C in S1 File). The age-adjusted incidence and multivariable-adjusted HR of ESRD were generally higher at lower levels of Hb, with more severe albuminuria, and at a lower eGFR (Fig 2, Table D in S1 File).

The multivariable-adjusted HRs associated with 1 g/dL lower Hb in participants with eGFR values ≥60, 30–59, and <30 mL/min/1.73 m^2^ were 1.34 (95% CI, 1.17–1.54), 1.55 (1.38–1.74), and 1.75 (1.47–2.09), respectively (*P*_interaction_ between eGFR groups = .057). In the analysis of 9 combined albuminuria-eGFR subgroups, HRs associated with 1 g/dL lower Hb were generally lower in persons with eGFR ≥60 than with <60 mL/min/1.73 m^2^, regardless of albuminuria categories (Table E in S1 File).

When biological interaction between low Hb, albuminuria, and eGFR groups was assessed using multivariable-adjusted HRs (Table D in S1 File) [17], the synergy index was 45.5. These results suggest a strong synergistic interaction.

## Discussion

Lower Hb levels were consistently associated with an increased risk of ESRD at a given severity of albuminuria and given levels of the eGFR. These results indicated that lower Hb interacts with albuminuria and a lower GFR to increase ESRD. Hb 13–13.9 g/dL in men, Hb 11–11.9 g/dL in women, and trace albuminuria by the dipstick test were associated with a more than doubled risk of ESRD after adjusting for confounders, including the eGFR. Women had stronger associations of low Hb and albuminuria with ESRD incidence than men. The HRs associated with lower Hb became stronger at ages ≥60 years than at ages <60 years in women.

### Anemia as a risk factor for CKD incidence in the general population without CKD

Anemia has been generally considered a complication of CKD and a risk factor for the progression of CKD, but it has not been generally recognized as a risk factor for CKD incidence in the general population [2,18], despite some suggestive evidence [19,20]. In our study, lower Hb was associated with an increased risk of ESRD at each category of albuminuria severity and eGFR, including the lowest risk group with an eGFR ≥60 mL/min/1.73 m^2^ and no albuminuria. These results suggest that lower Hb is a risk factor both for CKD incidence in the general population, as well as for CKD progression in CKD patients, potentially through kidney damage by anemia-induced renal hypoxia [19], although at least a part of lower Hb could be consequence of CKD. The correction of anemia in persons with anemia but without CKD might prevent CKD development; however, the current study could not examine the impact of anemia of various etiologies, and specific recommendations (e.g., for iron replacement therapy) are therefore beyond our scope.

### Stronger impact of anemia on CKD progression than on CKD incidence

In our study, people with albuminuria ≥trace or an eGFR <60 mL/min/1.73 m^2^ seemed to have stronger associations of lower Hb with ESRD incidence than people with no albuminuria or an eGFR ≥60 mL/min/1.73 m^2^. This may have been because the combined impacts of anemia and CKD in persons with CKD were stronger than the impact of anemia alone in persons without CKD.

### Clinical relevance of our findings: Surveillance

Anemia has not been recognized as a risk factor for recommendation of CKD screening. The prevalence of anemia was 10.9% in women and 3.5% in men in Korea [21]. In the US, the prevalence of anemia nearly doubled from 2003–2004 to 2011–2012 (4.0% to 7.1%)[22]. Hb measurements are a routine part of global health assessment in most adults [5]. Since lower Hb was strongly associated with ESRD incidence, when anemia in both sexes and Hb 13–13.9 g/dL in men are detected in health checkups, kidney function tests may be additionally performed in order to detect potential CKD. Moreover, since lower Hb without CKD was also associated with a higher risk of ESRD, further studies should investigate whether it is beneficial to routinely monitor for CKD and ESRD development in individuals with lower Hb but normal kidney function at the time of Hb measurements.

### Impact of Hb low-normal Hb levels and mild anemia

The current study demonstrated that men with Hb 13–13.9 g/dL had more than a doubled risk of ESRD compared with men with Hb ≥15 g/dL. Since Hb <13 g/dL is considered anemia in men, men with Hb 13–13.9 g/dL generally receive little attention in terms of the risk of CKD and ESRD. Furthermore, mild anemia in women (11–11.9 g/dL) and men (11–12.9 g/dL) is associated with over two-fold and four-fold increased risk of ESRD, compared with their respective counterparts. Proper management at earlier stage of anemia, for men with Hb 13–13.9 g/dL and individuals with mild anemia, might prevent CKD development in the general population and prevent progression to ESRD in CKD patients.

### Importance of trace albuminuria

In our study, trace albuminuria increased the ESRD risk by more than 3-fold in both women and men. Due to its limited accuracy, especially sensitivity (proportion of true positives [albuminuria] correctly identified) for detecting albuminuria, dipstick urinalysis receives relatively little attention compared to blood tests, and trace proteinuria detected by the dipstick test is often ignored. For example, in Korea and Japan, the national population screening programs use urinalysis, but trace proteinuria is not officially considered proteinuria [8,23]. Our results clearly indicated that individuals with dipstick trace proteinuria should not be ignored, but should be further evaluated for kidney damage. The dipstick test for proteinuria is a simple, inexpensive, and instantaneous test that can be easily performed in most clinical settings [24]. Since it has a good specificity (proportion of true negatives [no albuminuria] correctly identified) and negative predictive value (proportion of true negatives among test negatives), given the increased risk of ESRD associated with even trace proteinuria, the dipstick proteinuria test has a role for risk stratification, especially in resource-limited settings.

### Clinical relevance of our findings: Risk classification

The current study showed anemia (or, more broadly, Hb levels) to play a role in risk classification for the development and prognosis of CKD in the general population. As mentioned earlier, individuals with a low Hb (<14 g/dL in men or <12 g/dL in women), severe albuminuria, and GFR ≥60 mL/min/1.73 m^2^, who are considered a high-risk group, had a comparable risk to those with a GFR<30 mL/min/1.73 m^2^ but no albuminuria (the very high-risk group). Individuals with anemia (<13 g/dL in men or <11 g/dL in women), no albuminuria, and a GFR ≥60 mL/min/1.73 m^2^, who are considered a low-risk group by the KDIGO guidelines, had a comparable risk to those with a GFR 30–59 mL/min/1.73 m^2^, no albuminuria, and normal Hb (the moderate to high-risk group).

### Impact of anemia and low Hb in the elderly population

Many risk factors of diseases have weaker associations with diseases in older ages, as exemplified by the associations between BMI and mortality [6], blood pressure and cardiovascular disease [25], gamma glutamyl transferase and cardiovascular disease [26], and albuminuria and ESRD incidence in the current study. For ESRD incidence, the relative risks associated with anemia or lower Hb were not weaker in individuals aged ≥60 years than those aged <60 years in our study. In women, the risks associated with lower Hb were greater in older adults than in younger adults. As the prevalence of anemia increases with age in adult men and postmenopausal women, both the absolute and relative risks associated with lower Hb could be greater in the elderly, especially in women. Since life expectancy has increased worldwide and the prevalence of anemia is higher in older adults [27], the importance of the management of anemia or lower Hb may increase with advancing age for the prevention of CKD and ESRD.

### Strengths and limitations

The fact that Hb, rather than hematocrit, was used in the analysis is a strength of the study [5]. As a prospective cohort study, recall and selection biases related to the retrospective design were minimized. Nearly complete follow-up via record linkage to a national database is a further strength.

There are limitations in this study. First, the analysis of eGFR was limited to individuals who had a serum creatinine measurement during 2009–2010. Second, information on the etiology of anemia was unavailable. In Korea, more than half of anemias were iron deficiency anemia (IDA), while the proportion of IDA was markedly different according to sex and age [28]. The difference in the effect size of Hb by sex and age might be related to different etiology of anemia. Third, Hb and albuminuria assays were based on a single measurement. Although it is a limitation, using values that were measured once is less likely to overestimate the HRs due to regression dilution effects. Fourth, the study population comprised Koreans aged 40–80 years. Our results may not necessarily be generalized to younger people, or to other ethnic populations.

## Conclusions

Low-normal Hb levels and anemia were risk factors for ESRD incidence in general population without CKD as well as for the progression of CKD to ESRD. Lower Hb had synergic biological interactions with lower eGFR and albuminuria to increase the risk of ESRD incidence. Hb of 13–13.9 g/dL in men, 11–11.9 g/dL in women, and trace albuminuria by dipstick urinalysis were associated with a more than doubled risk of ESRD. The impacts of lower Hb may be stronger in older than younger women. The inclusion of persons with anemia or low-normal Hb in surveillance and management programs for the primary and secondary prevention of CKD may reduce the burden of CKD.

## Supporting information

S1 File Supplementary figures and tables. (PDF)
