# Supplementary material for: Low-normal hemoglobin levels and anemia are associated with increased risk of end-stage renal disease in general populations: A prospective cohort study
Source: PLoS One. 2019 Apr 25;14(4):e0215920. doi: 10.1371/journal.pone.0215920 (PMC6483202; doi:10.1371/journal.pone.0215920)
Supplement: S1 File — (PDF) [file pone.0215920.s001.pdf]

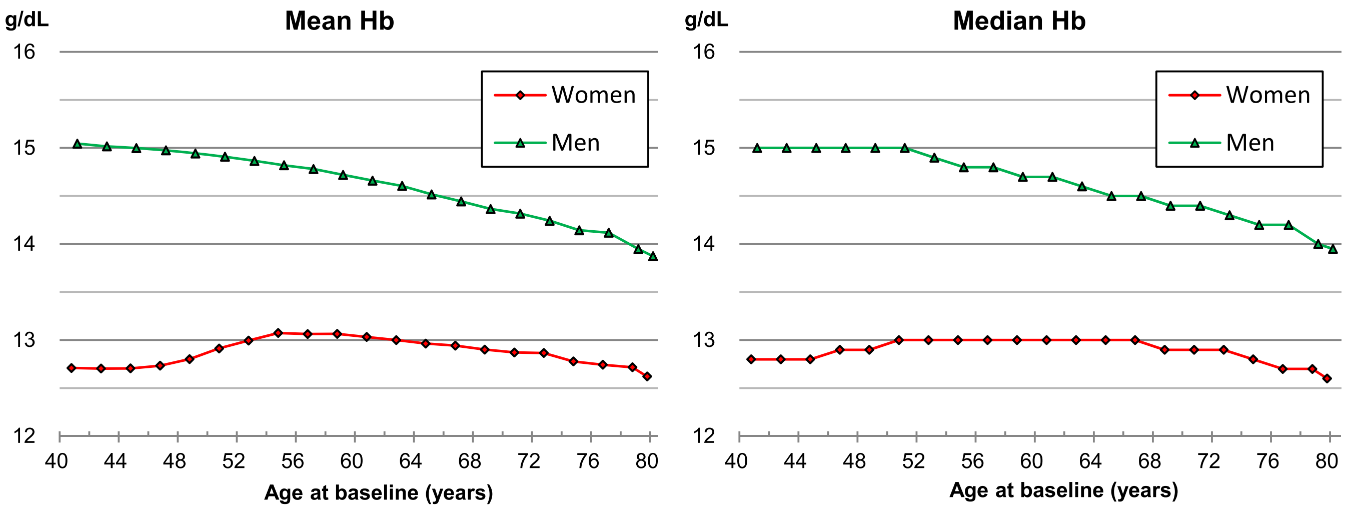

Figure A. Mean and median concentrations of hemoglobin by age and sex.

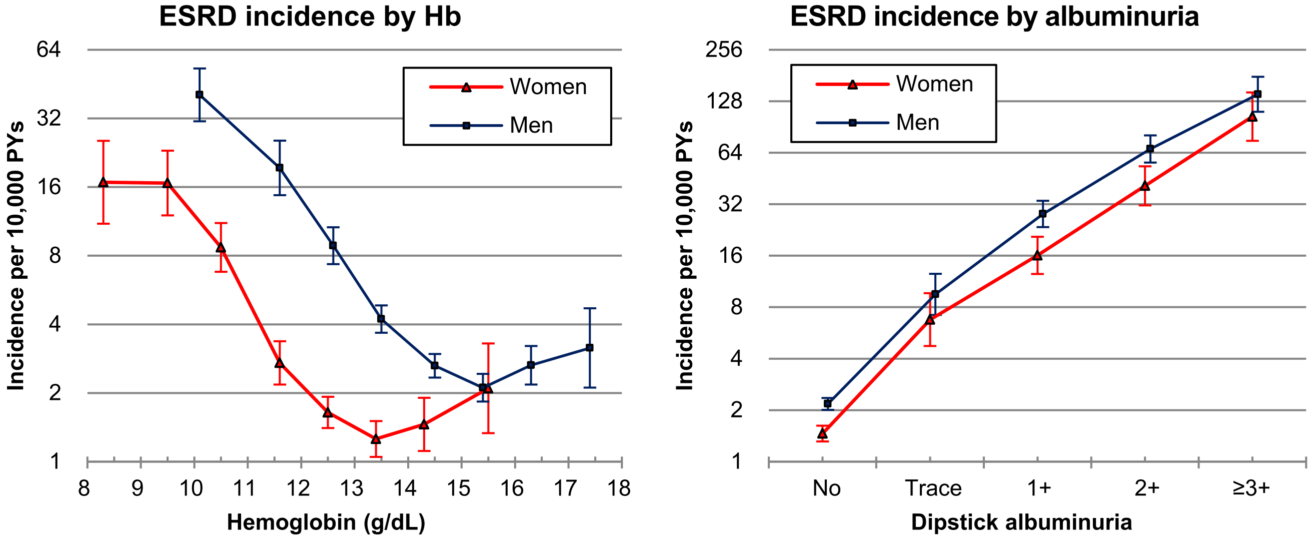

Figure B. Age adjusted* incidence of end stage renal disease (ESRD) by hemoglobin levels and severity of albuminuria according to sex.

PY, person-year. *Poisson regression was used.

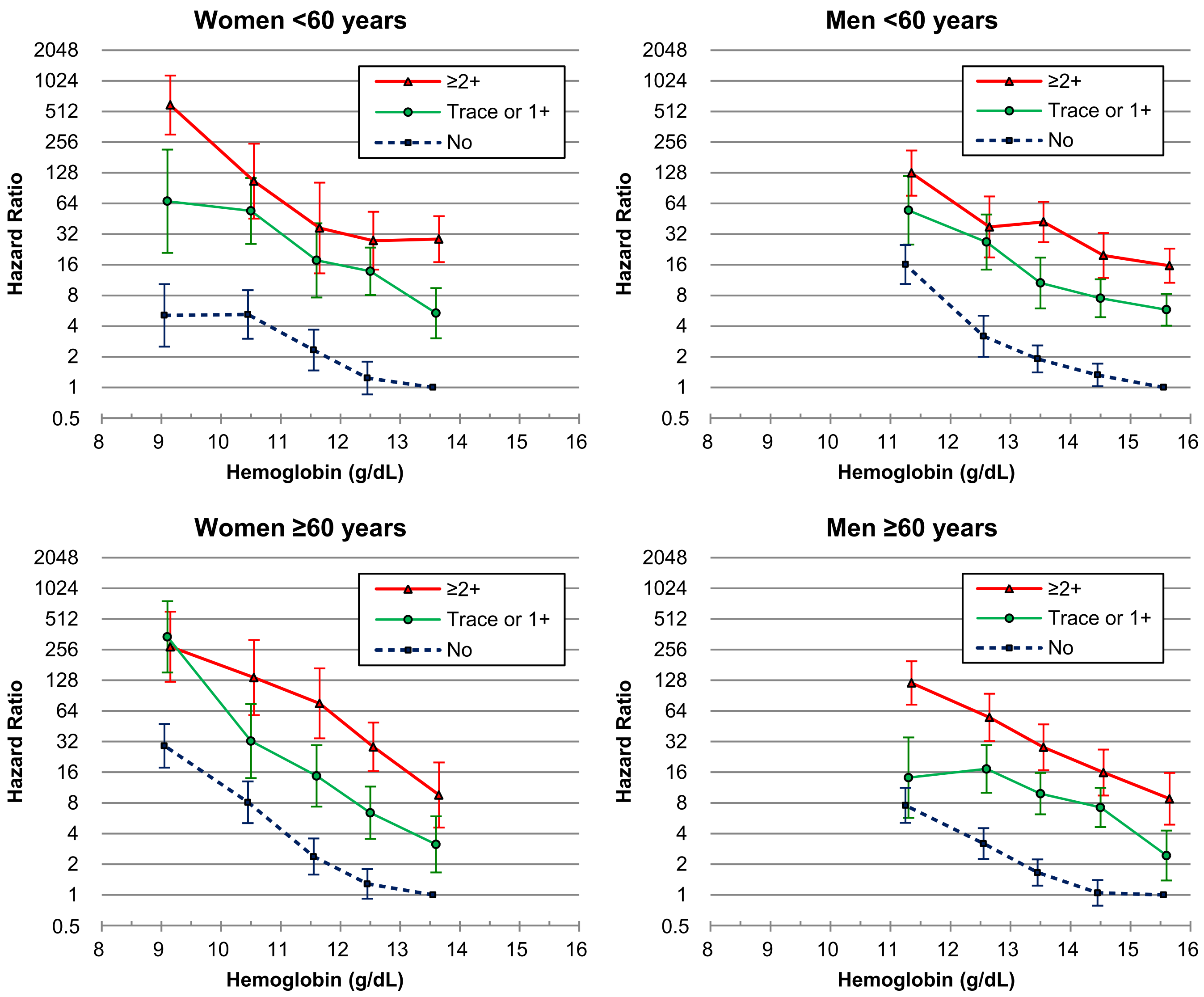

Figure C. HRs* for ESRD incidence in 15 combined hemoglobin and albuminuria groups in women and men according to age group.

ESRD, end-stage renal disease; HR, hazard ratio

*Adjustment for age at baseline, smoking status, alcohol use, body mass index, physical activity, income status, and dipstick hematuria, as well as comorbid diabetes, hypertension, cancer, and heart disease or stroke.

Table A. Characteristics of participants according to sex and albuminuria.

		Women					Men				
	Albuminuria	Subtotal	None	Mild	Severe		Subtotal	None	Mild	Severe	
Variables	Characteristics	N=233,497	n=225,173	n=6,860	n=1,464	*p*-value	N=277,123	n=267,344	n=7,897	n=1,882	*p*-value
Age	years	54.0 ±9.9	53.9±9.9	55.6±10.3	56.2±10.1	<.001	52.3 ±9.5	52.2±9.5	53.6±9.7	55.0±10.1	<.001
Hemoglobin (Hb)	g/dL	12.9 ±1.2	12.9±1.2	12.9±1.2	12.9±1.4	<.001	14.8 ±1.1	14.8±1.1	14.9±1.3	14.8±1.5	<.001
Total cholesterol	mg/dL	203 ±39	202±39	207±42	215±45	<.001	199 ±38	198±38	205±42	211±47	<.001
Smoking status	Never smoker	216,135 (92.6)	92.6	91.9	91.1	<.001	111,678 (40.3)	40.2	41.3	44.0	<.001
	Past smoker	2,152 (0.9)	0.9	0.8	1.9		41,262 (14.9)	14.9	15.6	14.9	
	Current smoker	6,398 (2.7)	2.7	3.2	3.7		111,450 (40.2)	40.3	38.8	37.1	
	Missing	8,812 (3.8)	3.8	4.1	3.3		12,733 (4.6)	4.6	4.3	4.0	
Alcohol use,	None	187,901 (80.5)	80.5	80.4	82.9	.176	95,540 (34.5)	34.4	35.6	37.9	<.001
times/week	<2	35,687 (15.3)	15.3	15.4	13.8		124,181 (44.8)	44.9	41.8	39.2	
	3-7	4,231 (1.8)	1.8	1.9	1.8		53,497 (19.3)	19.2	21.2	21.5	
	Missing	5,678 (2.4)	2.4	2.3	1.6		3,905 (1.4)	1.4	1.4	1.4	
Physical activity	≥1 times/week	74,782 (32.0)	32.0	32.1	33.5	.450	135,293 (48.8)	48.8	48.8	47.8	.685
Income status	<4 (low income)	67,202 (28.8)	28.8	27.0	27.7	.015	50,306 (18.2)	18.2	18.0	19.1	.002
(decile)	4-7	75,124 (32.2)	32.2	32.6	33.1		91,149 (32.9)	33.0	31.1	32.2	
	>7 (high income)	91,171 (39.0)	39.0	40.4	39.2		135,668 (49.0)	48.9	51.0	48.7	
Age group	<60 years	160,118 (68.6)	68.8	62.0	60.5	<.001	209,137 (75.5)	75.7	70.1	65.0	<.001
	≥60 years	73,379 (31.4)	31.2	38.0	39.5		67,986 (24.5)	24.3	29.9	35.0	
Albuminuria	No	225,173 (96.4)	100.0	0.0	0.0	<.001	267,344 (96.5)	100.0	0.0	0.0	<.001
	Trace	3,688 (1.6)	0.0	53.8	0.0		4,294 (1.5)	0.0	54.4	0.0	
	1+	3,172 (1.4)	0.0	46.2	0.0		3,603 (1.3)	0.0	45.6	0.0	

		Women					Men				
	**Albuminuria**	**Subtotal**	**None**	**Mild**	**Severe**		**Subtotal**	**None**	**Mild**	**Severe**	
	2+	1,157 (0.5)	0.0	0.0	79.0		1,460 (0.5)	0.0	0.0	77.6	
	≥3+	307 (0.1)	0.0	0.0	21.0		422 (0.2)	0.0	0.0	22.4	
Hematuria	No	204,064 (87.4)	88.2	67.2	63.4	<.001	264,861 (95.6)	96.1	83.8	74.7	<.001
	Trace	7,482 (3.2)	3.0	10.0	4.2		3,554 (1.3)	1.1	5.2	4.4	
	1+	12,225 (5.2)	5.0	12.4	8.7		5,324 (1.9)	1.7	6.5	7.7	
	2+	6,221 (2.7)	2.5	6.5	11.6		2,234 (0.8)	0.7	2.9	6.4	
	≥3+	3,505 (1.5)	1.4	4.0	12.2		1,150 (0.4)	0.3	1.6	6.9	
Comorbidity	Diabetes[a]	22,906 (9.8)	9.5	16.0	25.5	<.001	34,362 (12.4)	11.9	24.0	38.5	<.001
	Hypertension[b]	75,715 (32.4)	31.9	44.0	53.3	<.001	96,350 (34.8)	34.2	47.9	61.6	<.001
	Heart disease or stroke[c]	4,858 (2.1)	2.0	3.3	4.4	<.001	4,736 (1.7)	1.7	2.8	4.3	<.001
	Cancer[c]	1,604 (0.7)	0.7	0.7	0.7	>.999	1,258 (0.5)	0.4	0.7	0.7	.007

Data are expressed as mean±SD, n(%), or %. *P* values were calculated by chi-square test and one-way ANOVA between albuminuria groups.
Albuminuria was measured by dipstick test: None, mild (trace or 1+), severe (≥2+).
aPersons with known diabetes by hospital visit records or fasting glucose level ≥126 mg/dL at baseline health screening.
bPersons with known hypertension by hospital visit records or systolic blood pressure ≥140 mm Hg at baseline health screening.
cPersons with a history of the diseases via questionnaire

Table B. HRs[a] per 1 g/dL decrease in Hb for ESRD incidence by albuminuria categories

	Women				Men			
Albuminuria categories	No. of cases	*P*-value	HR per 1 g/dL lower Hb (95% CI)	$P_{Interaction}$ (albuminuria)	No. of cases	*P*-value	HR per 1 g/dL lower Hb (95% CI)	$P_{Interaction}$ (albuminuria)
No albuminuria	385	<.001	1.59 (1.47-1.71)	.086	699	<.001	1.50 (1.41-1.58)	.436
Trace	31	<.001	2.05 (1.54-2.73)		51	<.001	1.55 (1.28-1.87)	
1+	64	<.001	1.91 (1.60-2.27)		128	<.001	1.50 (1.32-1.70)	
2+	58	<.001	1.79 (1.49-2.13)		122	<.001	1.68 (1.51-1.87)	
≥3+	37	<.001	1.93 (1.56-2.40)		72	<.001	1.60 (1.34-1.92)	

CI, confidence interval; ESRD, end stage renal disease; Hb, hemoglobin; HR, hazard ratio; $P_{interaction}$ (albuminura), *P*-value for interaction between albuminuria groups

[a] Adjustment for age at baseline, sex, smoking status, alcohol use, physical activity, income status, diabetes, hypertension, a history of heart disease or stroke, a history of cancer, dipstick hematuria, total cholesterol, and body mass index.

Table C. Characteristics of participants with information of eGFR according Hb, albuminuria, and eGFR categories.

			Sex-specific Hb group			Dipstick albuminuria			eGFR		
	Hb group (g/dL)	Subtotal	Normal	Moderate	Low	None	Mild	Severe	≥60	30-59	10-29
Variables	Characteristics	N=349,993	n=274,695	n=53,657	n=21,641	n=332,258	n=14,337	n=3,398	n=323,740	n=25,840	n=413
Age	years	58.8 ±8.9	58.1±8.5	60.7±9.5	63.4±10.6	58.8±8.9	59.9±9.4	61.0±9.5	58.2±8.5	67.0±9.8	68.6±10.5
Hemoglobin (Hb)	g/dL	13.8 ±1.5	14.2±1.3	12.8±1.0	11.4±1.3	13.8±1.5	13.9±1.6	13.9±1.8	13.9±1.5	13.4±1.6	11.7±1.8
Body mass index	kg/m^2	24 ±2.9	24.2±2.9	23.6±2.9	23.3±2.9	24.0±2.9	24.5±3.1	24.9±3.2	24.0±2.9	24.4±3.0	24.4±3.1
Total cholesterol	mg/dL	200 ±38	201±38	198±39	195±40	200±38	204±39	207±41	200±38	205±39	206±43
Sex	Women	160,977 (46.0)	48.8	36.7	32.8	46.2	42.5	38.1	45.5	52.3	52.1
	Men	189,016 (54.0)	51.2	63.3	67.2	53.8	57.5	61.9	54.5	47.7	47.9
Smoking status	Never smoker	227,663 (65.0)	65.9	61.9	62.1	65.2	62.1	59.9	64.5	71.7	69.7
	Past smoker	30,163 (8.6)	8.3	9.8	9.8	8.6	9.1	8.6	8.7	7.4	9.0
	Current smoker	77,340 (22.1)	21.5	24.1	24.2	21.9	24.5	27.8	22.5	16.9	17.4
	Missing	14,827 (4.2)	4.3	4.2	3.9	4.2	4.3	3.7	4.3	4.0	3.9
Alcohol use,	None	191,748 (54.8)	55.2	52.9	54.5	54.9	52.7	51.9	53.9	65.3	64.9
times/week	<2	113,775 (32.5)	32.7	32.5	29.9	32.5	32.7	32.9	33.2	23.8	24.0
	3-7	38,074 (10.9)	10.2	12.9	14.0	10.8	12.7	13.4	11.0	8.9	8.7
	Missing	6,396 (1.8)	1.9	1.7	1.7	1.8	1.9	1.8	1.8	2.0	2.4
Physical activity	≥1 times/week	147,852 (42.2)	42.5	41.9	39.8	42.2	43.0	43.8	42.5	39.5	35.1
Income status	<4 (low income)	75,931 (21.7)	21.2	22.9	24.9	21.7	21.7	23.1	21.5	24.6	27.4
(decile)	4-7	114,059 (32.6)	32.1	34.0	35.0	32.6	31.8	32.9	32.6	31.9	37.3
	>7 (high income)	160,003 (45.7)	46.7	43.2	40.1	45.7	46.5	44.0	45.9	43.4	35.4
Albuminuria	No	332,258 (94.9)	95.1	94.9	92.8	100.0	0.0	0.0	95.3	90.3	65.6
	Trace	7,986 (2.3)	2.3	2.2	2.7	0.0	55.7	0.0	2.2	3.2	5.1

			Sex-specific Hb group			Dipstick albuminuria			eGFR		
	**Hb group (g/dL)**	**Subtotal**	**Normal**	**Moderate**	**Low**	**None**	**Mild**	**Severe**	**≥60**	**30-59**	**10-29**
	1+	6,351 (1.8)	1.7	1.8	2.6	0.0	44.3	0.0	1.7	3.6	13.3
	2+	2,577 (0.7)	0.7	0.8	1.2	0.0	0.0	75.8	0.6	2.0	10.2
	≥3+	821 (0.2)	0.2	0.3	0.6	0.0	0.0	24.2	0.2	0.9	5.8
Hematuria	No	322,284 (92.1)	92.1	92.1	92.3	92.2	89.9	86.8	92.2	90.2	87.2
	Trace	7,421 (2.1)	2.1	2.1	2.1	2.1	2.5	2.8	2.1	2.6	3.4
	1+	11,607 (3.3)	3.3	3.3	3.0	3.3	4.1	4.6	3.3	4.1	5.1
	2+	5,570 (1.6)	1.6	1.7	1.6	1.5	2.4	3.2	1.6	1.9	2.9
	≥3+	3,111 (0.9)	0.9	0.9	1.1	0.9	1.2	2.6	0.9	1.2	1.5
Comorbidity	Diabetes[a]	33,788 (9.7)	8.8	11.6	15.2	9.2	16.1	28.1	9.1	16.7	36.1
	Hypertension[b]	108,494 (31.0)	30.2	32.7	37.4	30.4	40.8	52.1	29.4	50.1	67.3
	Heart disease or stroke[c]	5,322 (1.5)	1.4	1.7	2.5	1.5	2.3	3.1	1.3	3.9	5.6
	Cancer[c]	1,685 (0.5)	0.4	0.5	1.2	0.5	0.4	0.4	0.5	0.7	0.5
eGFR,	≥60	323,740 (92.5)	93.6	90.3	84.0	92.9	87.1	76.2	100.0	0.0	0.0
mL/min/1.73 m^2	30-59	25,840 (7.4)	6.4	9.5	15.0	7.0	12.4	21.9	0.0	100.0	0.0
	10-29	413 (0.1)	0.04	0.2	1.0	0.1	0.5	1.9	0.0	0.0	100.0

eGFR, estimated glomerular filtration rate; Hb hemoglobin;
Data are expressed as mean±SD, n(%), or %.
P values which were calculated by chi-square test and one-way ANOVA between Hb, albuminuria, and eGFR groups, were <.001 for each variable except for hematuria between Hb group (*p*=.009) and physical activity (*p*=.032), income status (*p*=.039), and comorbid cancer (*p*=.633) between albuminuria group.
aPersons with diabetes or fasting glucose level ≥126 mg/dL at baseline health screening.
bPersons with hypertension or systolic blood pressure ≥140 mm Hg at baseline health screening.
cPersons with a history of the diseases via questionnaire

Table D. HRs for ESRD incidence by 27 Hb, albuminuria and eGFR combined group

eGFR (mL/min/ 1.73 m^2)	Albuminuria (dipstick)	Sex-specific Hb group	No. of subjects	No. of cases	Age and sex adjusted		Multivariable adjusted[a]	
					P-value	HR (95% CI)	*P*-value	HR (95% CI)
≥60	None	Normal	245,241	46		1.00 (Reference)		1.00 (Reference)
		Moderate	46,286	16	.051	1.76 (1.00-3.12)	.064	1.72 (0.97-3.04)
		Low	17,135	15	<.001	4.58 (2.54-8.23)	<.001	4.28 (2.37-7.71)
	Trace or 1+	Normal	9,855	8	<.001	4.29 (2.02-9.09)	<.001	3.66 (1.73-7.78)
		Moderate	1,793	6	<.001	16.94 (7.22-39.75)	<.001	13.57 (5.77-31.92)
		Low	842	3	<.001	18.94 (5.87-61.05)	<.001	14.70 (4.55-47.51)
	≥2+	Normal	1,992	6	<.001	15.64 (6.68-36.64)	<.001	11.12 (4.73-26.16)
		Moderate	392	6	<.001	77.31 (32.93-181.52)	<.001	50.00 (21.13-118.30)
		Low	204	4	<.001	105.15 (37.71-293.17)	<.001	59.59 (21.19-167.54)
30-59	None	Normal	15,932	12	<.001	3.89 (2.04-7.39)	<.001	3.39 (1.78-6.46)
		Moderate	4,581	10	<.001	11.17 (5.57-22.39)	<.001	8.80 (4.38-17.69)
		Low	2,812	27	<.001	51.95 (31.50-85.68)	<.001	37.93 (22.89-62.85)
	Trace or 1+	Normal	1,137	5	<.001	22.54 (8.91-57.05)	<.001	16.75 (6.60-42.52)
		Moderate	357	4	<.001	59.07 (21.09-165.45)	<.001	41.23 (14.67-115.90)
		Low	277	18	<.001	361.16 (205.82-633.75)	<.001	222.23 (125.26-394.27)
	≥2+	Normal	426	16	<.001	199.69 (112.23-355.31)	<.001	125.09 (69.51-225.12)
		Moderate	160	15	<.001	534.39 (295.38-966.80)	<.001	286.26 (156.13-524.84)
		Low	158	21	<.001	791.87 (465.13-1348.15)	<.001	382.51 (220.63-663.16)
10-29	None	Normal	84	1	<.001	68.23 (9.38-496.14)	<.001	59.01 (8.10-429.80)
		Moderate	59	2	<.001	210.97 (50.73-877.26)	<.001	139.73 (33.45-583.74)
		Low	128	15	<.001	808.78 (442.44-1478.44)	<.001	441.19 (238.65-815.65)
	Trace or 1+	Normal	19	4	<.001	1274.83 (455.07-3571.32)	<.001	817.37 (288.95-2312.12)
		Moderate	13	2	<.001	966.37 (233.09-4006.48)	<.001	600.22 (143.46-2511.35)
		Low	44	19	<.001	3696.13 (2116.59-6454.42)	<.001	1886.55 (1061.35-3353.35)
	≥2+	Normal	9	2	<.001	1347.92 (325.22-5586.57)	<.001	1076.22 (256.48-4515.95)
		Moderate	16	7	<.001	3611.61 (1612.47-8089.26)	<.001	1517.84 (655.80-3513.02)
		Low	41	26	<.001	5827.57 (3569.50-9514.09)	<.001	3247.64 (1947.95-5414.49)

CI, confidence interval; eGFR, estimated glomerular filtration rate; ESRD, end stage renal disease: Hb, hemoglobin; HR, hazard ratio
Hb categories (g/dL): ≥12 (normal), 11-11.9 (moderate), <11 (low) in women; ≥14 (normal), 13-13.9 (moderate), <13 (low) in men.
a Adjustment for age at baseline, sex, smoking status, alcohol use, physical activity, income status, diabetes, hypertension, a history of heart disease or stroke, a history of cancer, dipstick hematuria, total cholesterol, and body mass index.

Table E. HRs[a] per 1 g/dL decrease in Hb for ESRD incidence by albuminuria and eGFR combined categories

eGFR categories	Albuminuria categories	No. of cases	*P*-value	HR per 1 g/dL lower Hb (95% CI)	$P_{Interaction}$ (albuminuria)	$P_{Interaction}$ (albuminuria and eGFR)
≥60	No albuminuria	77	0.005	1.29 (1.08-1.53)	.628	.004
	Trace or 1+	17	0.009	1.54 (1.11-2.13)		
	≥2+	16	0.067	1.31 (0.98-1.75)		
30-59	No albuminuria	49	<0.001	2.01 (1.70-2.38)	.227	
	Trace or 1+	27	<0.001	1.85 (1.45-2.35)		
	≥2+	52	<0.001	1.61 (1.34-1.94)		
10-29	No albuminuria	18	0.004	1.76 (1.20-2.57)	.214	
	Trace or 1+	25	0.002	1.81 (1.24-2.65)		
	≥2+	35	<0.001	2.79 (1.83-4.26)		

CI, confidence interval; eGFR, estimated glomerular filtration rate; ESRD, end stage renal disease; Hb, hemoglobin; HR, hazard ratio; $P_{interaction}$ (albuminura), *P*-value for interaction between albuminuria groups

[a] Adjustment for age at baseline, sex, smoking status, alcohol use, physical activity, income status, diabetes, hypertension, a history of heart disease or stroke, a history of cancer, dipstick hematuria, total cholesterol, and body mass index.
